# Molecular and Computational Methods for the Detection of Microsatellite Instability in Cancer

**DOI:** 10.3389/fonc.2018.00621

**Published:** 2018-12-12

**Authors:** Laura G. Baudrin, Jean-François Deleuze, Alexandre How-Kit

**Affiliations:** ^1^Laboratoire de Génomique, Fondation Jean Dausset–CEPH, Paris, France; ^2^Laboratoire d'Excellence GenMed Paris, Paris, France; ^3^Centre National de Recherche en Génomique Humaine, CEA–Institut François Jacob, Evry, France

**Keywords:** microsatellite instability, MSI-H cancer, MSI detection method, next-generation sequencing, computational biology, microsatellite genotyping

## Abstract

Microsatellite instability (MSI) is a genomic alteration in which microsatellites, usually of one to four nucleotide repeats, accumulate mutations corresponding to deletions/insertions of a few nucleotides. The MSI phenotype has been extensively characterized in colorectal cancer and is due to a deficiency of the DNA mismatch repair system. MSI has recently been shown to be present in most types of cancer with variable frequencies (from <1 to 30%). It correlates positively to survival outcome and predicts the response to immune checkpoint blockade therapy. The different methods developed for MSI detection in cancer require taking into consideration two critical parameters which influence method performance. First, the microsatellite markers used should be chosen carefully to ensure they are highly sensitive and specific for MSI detection. Second, the analytical method used should be highly resolute to allow clear identification of MSI and of the mutant allele genotype, and should present the lowest limit of detection possible for application in samples with low mutant allele frequency. In this review, we describe all the different molecular and computational methods developed to date for the detection of MSI in cancer, how they have evolved and improved over the years, and their advantages and drawbacks.

## Introduction

Microsatellite instability (MSI/MSI-H) is characterized by the accumulation of mutations (insertions or deletions of a few nucleotides) in microsatellites (also known as short tandem repeats), which are continuous repetitions of 1–9 nucleotides. It has been shown that MSI is caused by loss of function of a member of the DNA mismatch repair system (MMR), which normally allows the repair and correction of DNA following DNA mismatches introduced by DNA polymerase (1). The MMR system comprises at least ten proteins including MLH1, MSH2, MSH6, and PMS2, which are the most frequent mutated or epimutated (*MLH1*) genes in cancer (1–6).

MSI has been described and extensively characterized in colorectal cancer in which 15–20% of tumors present the MSI phenotype which is correlated with better patient survival (7–9). In addition, 3% of MSI colorectal cancers arise in the context of an inherited syndrome called Hereditary Non-Polyposis Colorectal Cancer (HNPCC) or Lynch syndrome, in which a constitutional mutation of an MMR gene leads to an increased risk of cancer incidence requiring specific management (10–12). A second, more severe syndrome called Constitutional Mismatch Repair Deficiency (CMMRD) is due to bi-allelic germline mutations of one of the four MMR genes (*MLH1, MSH2, MSH6*, or *PMS2*), and is characterized by the appearance of multiple neoplasia including colorectal cancer in childhood (13, 14).

MSI has also been detected in other types of cancer including gastric (6), endometrial (5), ovarian (15), and liver (16) cancer, however it is only recently that MSI has been widely identified across several types of cancer through studies based on the analysis of whole genome/exome sequencing data (4, 17, 18). The incidence of MSI in cancer correlates positively to survival outcome (17) and can also predict the efficacy of immune checkpoint blockade therapy in solid tumors (19, 20).

Different approaches have been developed for MSI detection in cancer with the aim of providing the highest degree of sensitivity and specificity. The first parameter influencing the detection of MSI in cancer is the type of microsatellite markers used. Different microsatellites and microsatellite panels have been proposed for sensitive detection of MSI including the Bethesda/NCI panel, which has been the gold standard microsatellite panel for MSI detection for more than 20 years (21–23). This gold standard method relies principally on PCR followed by capillary electrophoresis fragment analysis, whereas other methods also developed to improve the limit of detection (LOD) of MSI, as required for some applications (e.g., to detect MSI in blood and in tumors with a high level of normal cell contamination or in precancerous lesions), rely on the modification of standard procedures (24–26). Recently, with the development of next-generation sequencing (NGS), new computational algorithms have emerged which allow the detection of MSI in thousands of microsatellite markers, a discovery which could change the standard of MSI detection in cancer (27–29).

In this review, we describe the different approaches developed to date for the detection of MSI in cancer, and we highlight and compare the advantages and drawbacks of each method.

## Evolution of the Standard Technologies for MSI Detection and Identification in Cancer

**Figure 1:**
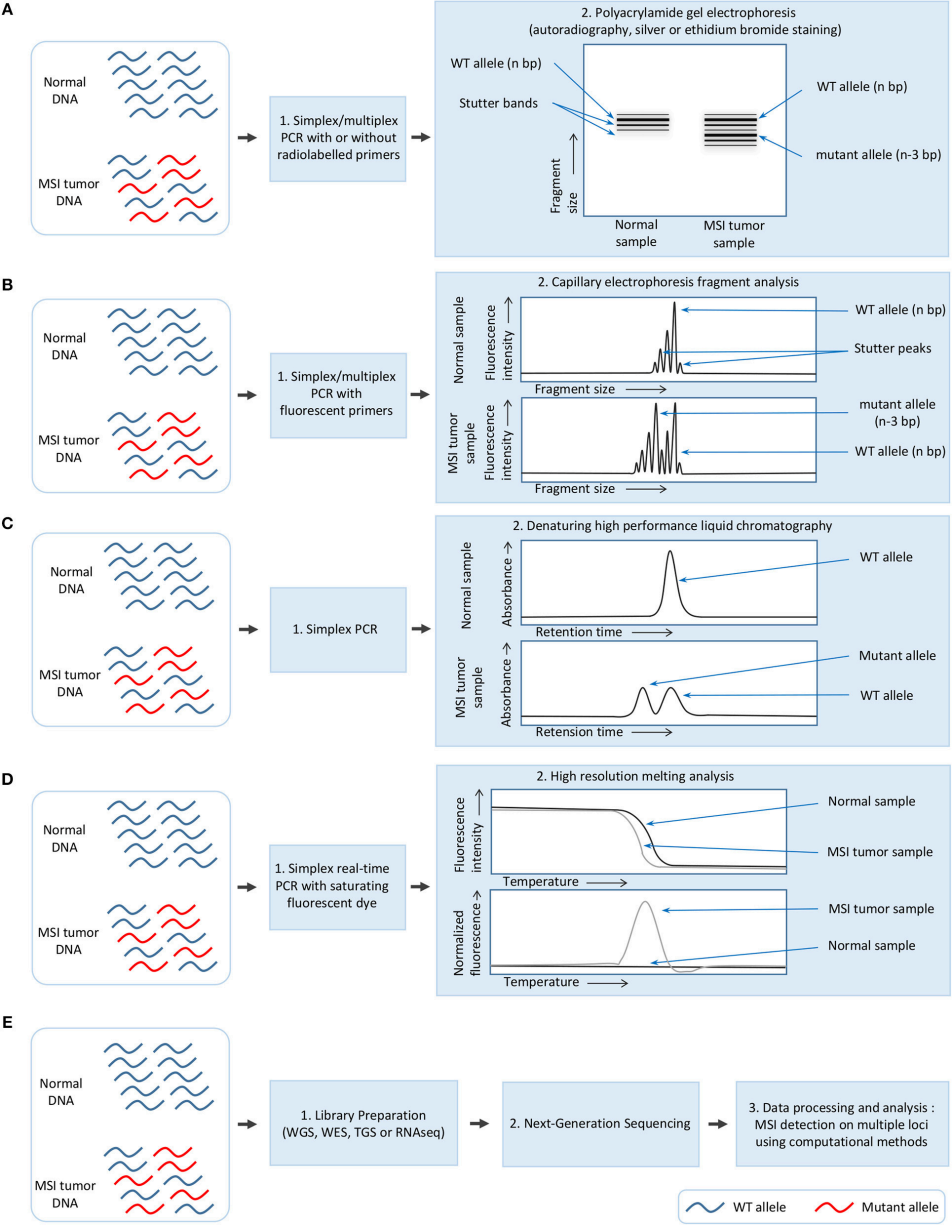
Overview of the different methods used for MSI detection in cancer. **(A)** Polyacrylamide gel electrophoresis (PAGE). **(B)** Capillary electrophoresis fragment analysis (FA). **(C)** Denaturing high performance liquid chromatography (DHPLC). **(D)** High resolution melting analysis (HRM). **(E)** Next-generation sequencing (NGS).

Although the methods used for MSI detection in cancer have constantly evolved, they still rely on two constants: (i) the amplification of one or several microsatellite markers with PCR-based methods, and (ii) the detection of MSI by methods which allow fragment length analysis (Figure 1).

MSI detection was initially performed in colorectal cancer either by using PCR on specific markers followed by polyacrylamide gel electrophoresis and autoradiography (7, 30), or a fingerprinting method based on arbitrarily primed PCR (AP-PCR) in which primers with arbitrarily chosen sequences are used in a PCR characterized by a few low stringency cycles followed by several higher stringency cycles, and combined with electrophoresis for fragment separation (9, 31). In some laboratories, MSI detection was performed with silver or ethidium bromide staining of polyacrylamide gel after electrophoresis rather than autoradiography (Figure 1A) (32).

These methods, which proved laborious and time-consuming and presented a low sizing accuracy, were supplanted by a newer approach which remains today the gold standard for MSI detection. This approach combines a PCR with fluorescent primers and capillary electrophoresis using a DNA sequencer which allows fragment analysis at single base resolution (Figure 1B) (33). This procedure has also been improved by the multiplexing of PCR to allow amplification of 2–5 microsatellite markers (34–40) and automatic identification of the allele size (34).

Other read-out methods have also been used for MSI detection following PCR amplification of microsatellite markers, including denaturing high-performance liquid chromatography (DHPLC) (41–43) and high resolution melting (HRM) analysis (44). DHPLC relies on the separation of DNA strands based on the size and sequence, and presents the advantage of being free from stutter peaks [undesirable frameshift products, multiples of the repeated nucleotide sequence, and generally shorter than the specific amplification product, which are formed due to slippage of DNA polymerase during PCR amplification (45)]; DHPLC has a limit of detection of 1% of mutated alleles (Figure 1C) (41). In contrast, HRM is a rapid, closed-tube post-PCR method based on the slow denaturation of the PCR products and the differences in the denaturation curves between mutated and wild-type; its drawback is that it allows the detection but not the identification of MSI (Figure 1D) (44).

Recently, massive parallel next-generation sequencing (NGS) has also been used to detect MSI in cancer (4, 17), with a limit of detection evaluated at 1% of MSI in an MSS background (Figure 1E) (46). The great advantage of NGS is that MSI can be simultaneously detected in a large number of microsatellite loci, ranging from five to several hundred thousand depending on the protocol used for library preparation (4, 27, 47). Library preparation is based on the standard procedures for whole genome sequencing (WGS), whole exome sequencing (WES) and targeted gene sequencing (TGS) of gene panels [e.g., ColoSeq, UW-OncoPlex (27), BROCA (3), ColonCore NGS panel (48), and smMIP panel (46)]; this last approach is based either on amplicon sequencing (49), complementary RNA (cRNA) biotinylated oligonucleotide sequence capture (50) or single-molecule molecular inversion probe capture (51). A proof of concept study using ultra-deep sequencing has also been performed using the Bethesda/NCI panel. Five markers were sequenced with a coverage of 5000–8000 x and gave similar results to the gold standard method, thus demonstrating that NGS can be used for MSI testing (47). However, MSI detection by NGS requires the development of specific algorithms and computational methods which will be described later in this review.

## The Choice of Microsatellite Markers Improves the Sensitivity of MSI Detection in Cancer

MSI was initially described in 1993 in colorectal cancers in which deletions in several amplicons of mono-, di-, and tri-nucleotide repeats were shown to be present in 12% of the tumors, using an AP-PCR approach (9). The same year, two other studies detected MSI in sporadic (13–28%) and hereditary non-polyposis (79%) CRC, based on PCR and gel electrophoresis using four dinucleotide (CA) microsatellite markers (Mfd 26, Mfd 27, Mfd 41, and 635/636) (7) or 6 dinucleotide (CA) microsatellite markers (D2S119, D2S123, D2S147, D10S197, D11S904, and D13S175) and one trinucleotide (CTG) microsatellite marker (CTG-B37) (30). The percentage of tumors with MSI, based on each marker taken alone, varied greatly indicating that all microsatellites present different sensitivities for MSI detection as they probably display different stabilities for MMR deficiency (2, 30, 32, 33, 52). Thus, a combination of the most sensitive microsatellite markers should be used for MSI assessment in cancer.

In order to standardize the panel of microsatellites used for MSI detection in cancer across the different laboratories in the world, a National Cancer Institute (NCI) workshop held in Bethesda, (Maryland, USA) proposed a panel of two mononucleotide (BAT-25 and BAT-26) and three dinucleotide (D2S123, D5S346, and D17S250) repeat microsatellites called the Bethesda/NCI panel for the detection of MSI in colorectal cancer, and according to which tumors presenting two or more unstable markers (or ≥30–40% if more markers are tested) should be defined as MSI/MSI-H (21). The other tumors are classified as MSS (microsatellite stable) or MSI-L (MSI-low) if no markers or only one marker is unstable (or <30–40% if more markers are tested) (21). Though still under debate, MSS and MSI-L tumors are usually considered and treated clinically as a single group, and it is generally agreed today that MSS and MSI-L tumors belong to the same group, as most MSS tumors can normally be classified as MSI-L if a sufficient number of microsatellite markers are tested (17, 23, 53–56).

Although the Bethesda/NCI panel is recommended again in the revised Bethesda guidelines for Lynch syndrome and MSI (22), and remains today the gold standard used in several laboratories for MSI detection, several concerns have arisen regarding this panel (57, 58). These concerns notably relate to the presence of dinucleotide markers in the panel, which have a lower ability to detect MSI-H tumors due to their lower sensitivity compared to mononucleotide microsatellites, and they require matched normal samples for MSI detection due to their natural polymorphism in individuals, which can lead to MSS/MSI-L tumors being misclassified as MSI-H (58–63).

Therefore, some researchers have claimed that mononucleotide repeat microsatellites are the most appropriate markers for MSI detection, as they are more sensitive and almost monomorphic, thus requiring less use of matched normal samples (57, 58, 60, 62, 63). The use of BAT-26, a quasi-monomorphic microsatellite present in Caucasian but not in African populations, has been proposed for MSI detection (with no need to match normal DNA), either on its own (57, 60) or in combination with BAT-25 (64), and has proved effective in large cohort studies (10, 65). In 2002, the same authors proposed a set of five quasi-monomorphic mononucleotide repeat microsatellites (BAT-25, BAT-26, NR-21, NR-22, and NR-24), known as the pentaplex panel, which allow the detection of MSI without matching normal DNA, and offer better performance compared to BAT-26 or BAT-25 alone (35), or compared to the Bethesda panel (66). Most of these markers have been combined in a new mononucleotide repeat pentaplex panel (NR-21, NR-24, BAT-25, BAT-26, and NR-27/MONO-27), optimized for MSI detection based on the screening of 266 mono- to penta-nucleotide repeat microsatellites. This panel has out-performed the current Bethesda panel (36, 37, 63, 67), and is currently marketed as the MSI Analysis System by Promega Corporation (Madison, Wisconsin, USA) and is also considered as a gold standard panel for MSI detection in cancer.

Several other mononucleotide repeat microsatellites have been proposed as sensitive and specific markers for MSI detection in cancer. Among these are CAT-25, a quasi-monomorphic mononucleotide repeat microsatellite in all populations, which has been put forward as a single marker for MSI detection in CRC, offering improved performance compared to the Bethesda panel, BAT-25 or BAT-26 (68, 69). CAT-25 has also been proposed in combination with three mononucleotide repeat microsatellites (BAT26, NR21, and NR27) in a tetraplex panel which allows MSI detection in solid tumors, notably in MSH6-deficent tumors where the pentaplex panel fails at times to detect MSI (38). Another microsatellite, HT-17, has also been proposed as a sole marker for MSI detection in CRC as it presents better sensitivity compared to the pentaplex panel (70, 71). Moreover, HT-17 has been found to carry prognostic and predictive information on the response to chemotherapy (5-FU and oxaliplatin) in CRC patients where large deletions in HT-17 indicate a better prognosis and better response to treatment (70, 72). Recently, a panel of five long mononucleotide repeat microsatellites (BAT-52, BAT-55, BAT-56, BAT-57, and BAT-59), also evaluated for MSI detection in early colorectal lesions, showed improved sensitivity compared to the Bethesda and pentaplex panels (73).

Since the development of NGS, a greater number of microsatellites, and potentially all microsatellites in the genome, can now be analyzed for MSI detection, an advance which could further improve the detection of MSI in cancer. However, two studies based on WGS and WES NGS data have shown that most microsatellites in the genome are stable and therefore would not be informative in the case of MMR deficiency (4, 17). These studies also showed that MSI frequently affects mononucleotide repeat microsatellites, in particular those of 16 repeat units in length as well as microsatellites located in intergenic, intronic, and 3′UTR regions (4, 17). Both studies demonstrated that some unstable microsatellites were intra- and/or inter-tumor-type specific, and also identified the most frequently recurring unstable microsatellites across all MSI-H cancers, a finding which could further be used to define new pan-cancer panels for MSI detection with improved sensitivity and specificity (4, 17). The development of new specific cancer-type and pan-cancer panels could be useful especially as the microsatellites in the Bethesda and pentaplex panels, which are principally recommended for CRC but used in all types of malignancies, have shown poor performance in other types of cancer (46, 74–76).

To date, most studies on MSI detection in cancer that are based on NGS experiments have either evaluated MSI in several types of microsatellites (mono- to penta-nucleotide repeat) or refined their analysis to mononucleotide repeat microsatellites using microsatellite sequencing data available on WGS, WES or TGS of panels of genes implicated in cancer which were not initially designed for MSI detection (4, 17, 18, 27, 28, 56). Only three panels recently published are specifically designed for MSI detection in cancer, namely the MSIplus panel which evaluates mutations in 3 oncogenes (KRAS, BRAF, and NRAS) and 17 microsatellites (49), the ColonCore Panel which allows the simultaneous detection of MSI and mutations in 36 CRC-related genes principally proposed for CRC (48), and a pan-cancer panel of 111 microsatellite loci highly informative in cancer (46). These panels have shown either a comparable or better performance for MSI detection in colorectal and non-colorectal cancers than the pentaplex panel (46).

## Improving the Limit of Detection of MSI in Cancer Through Modification of the Standard Protocol

Capillary electrophoresis fragment analysis is the standard molecular method for MSI detection in cancer, however this method presents a LOD which might not be sufficient for some clinical applications such as the detection of MSI in blood, plasma, precancerous lesions, and tumors with high levels of normal cell contamination, or in tumor heterogeneity where only a small subset of the tumor cells present MSI. The particularity of the LOD of MSI is that it can vary (from 1 to 10%) according to the length of the mutant alleles and the WT genotype, so that large deletions present a lower LOD than small deletions due to the presence of stutter peaks which mask mutant alleles (26).

**Figure 2:**
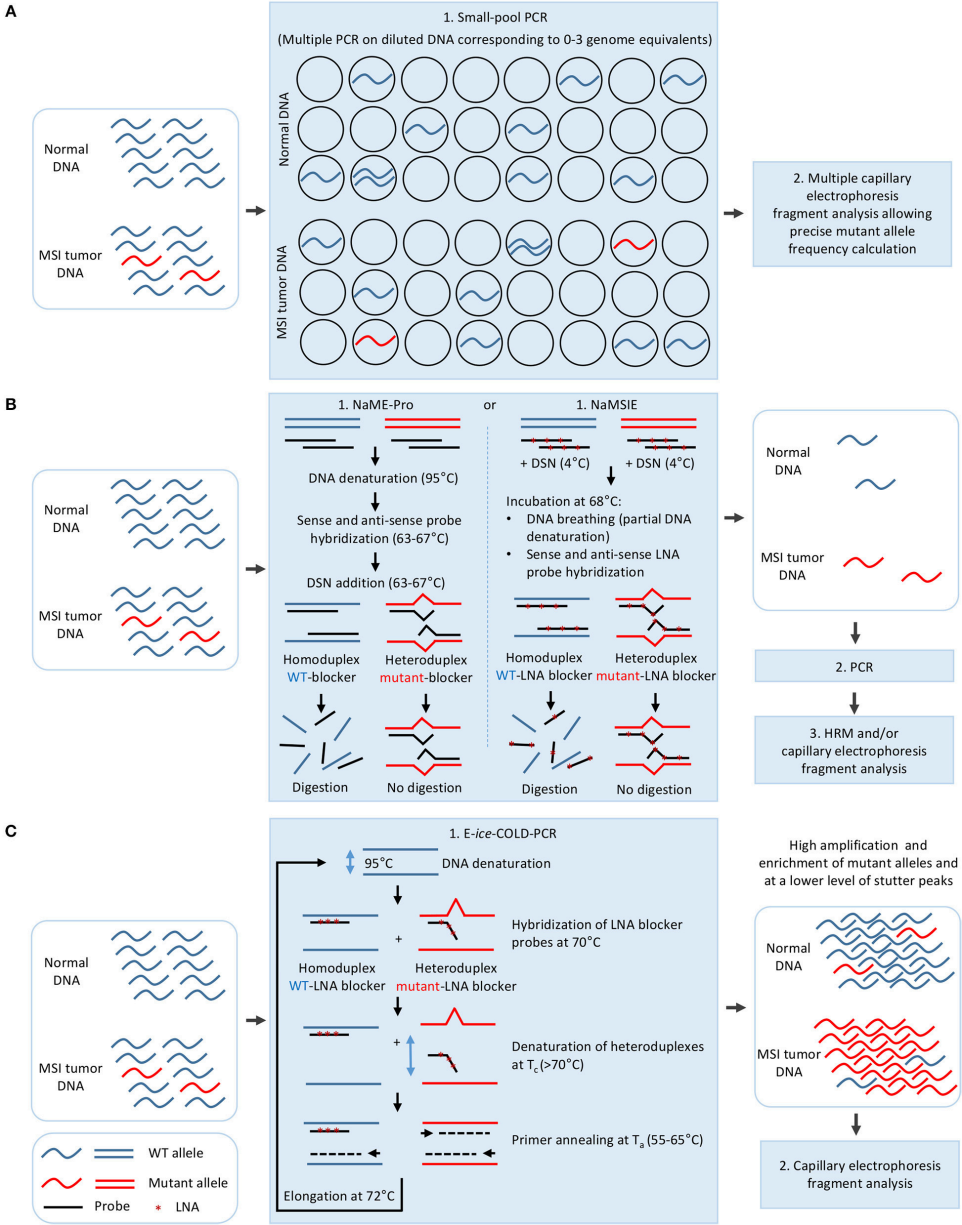
Strategies for improving the limit of detection of MSI in cancer. **(A)** Dilution of the DNA to 0–3 genome equivalent per PCR in small-pool PCR. **(B)** Mutant allele enrichment in the DNA sample using sense and antisense probes complementary to WT DNA sequence and duplex specific nuclease (DSN) in NaME-Pro and NaMSIE. **(C)** Mutant allele enrichment during E-*ice*-COLD-PCR using an LNA blocker probe complementary to WT DNA sequence. LNA, Locked nucleic acid.

Therefore, different approaches which modify a single step of the standard fragment analysis protocol have been developed to improve the LOD of MSI (Figure 2). A first such approach proposed for diagnosis of CMMRD syndrome was based on MSI detection in lymphoblastoid cell lines derived from CMMRD patients rather than in blood, and which had developed *ex vivo* MSI phenotype during *in vitro* culture (77). While this method presents a clinical sensitivity and specificity of 100%, the time to result is 120 days after immortalization (77).

Three other methods have been proposed for MSI detection, all of which rely on a pre-PCR modification of the standard procedure. In small-pool PCR, the DNA is diluted so that the PCR is performed on 0–3 genome equivalents, thus allowing the mutated alleles to be easily detected and quantified (Figure 2A) (78–81). This method has allowed MSI detection in non-tumoral colon mucosa, urinary tract epithelial cells, PBL, saliva, and lymphoblastoid cell lines of HNPCC patients, however it requires multiple replicate PCR experiments (at least a hundred PCRs with one genome equivalent for a limit of detection of 1%) (80–82). Nuclease-assisted minor allele enrichment with either probe overlap (NaME-Pro) and nuclease-assisted microsatellite instability enrichment (NaMSIE) are two recently developed methods based on the use of DNA and locked-nucleic-acid(LNA)-containing probes complementary to WT microsatellite sequences, respectively, combined to the preferential digestion of fully matched over mismatch-containing double-stranded DNA by a duplex-specific nuclease (DSN), which leads to the enrichment of mutant alleles prior to PCR (Figure 2B) (24, 83). These methods present a limit of detection of 0.5 and 0.01% of mutant alleles when combined to capillary electrophoresis and HRM, respectively, and greatly improve MSI detection in CRC samples with low tumoral cell content and circulating DNA of plasma samples of CRC patients (24, 83).

One such method proposed to improve MSI detection and derived from a modification of the PCR protocol, is based on E-*ice*-COLD-PCR, a method previously developed for enrichment of point mutations located in mutational hotspots in oncogenes such as *BRAF, NRAS*, and *KRAS* (84–88). This method relies on the use of a non-elongable poly T LNA blocker probe complementary to the WT sequence which hybridizes to the WT and mutant sequence, and on the preferential denaturation of the mutant-probe heteroduplex compared to WT-probe homoduplex at a critical temperature (Tc) to allow a strong enrichment of mutant microsatellite alleles during primer annealing/elongation steps (Figure 2C) (26). E-*ice*-COLD-PCR lowers the limit of detection of MSI to 0.05% of mutant alleles, allowing MSI detection in 100% of CRC samples with a high level of normal cell contamination, however it requires the use of WT control samples due to the enrichment of stutter peaks during PCR amplification (26).

Finally, a post-PCR method called gMSI has been developed to improve the sensitivity of MSI detection in blood from CMMRD patients. This method relies on the analysis of the height of n+1 and n+2 stutter peaks compared to the n peak (where n is the size of the WT allele) and uses freely available software (25). Although gMSI is more sensitive than the standard method, it is unable to detect MSI due to MSH6 deficiency (77).

## Computational Methods for MSI Detection in Cancer

Before the advent of NGS, to our knowledge only two mathematical prediction models had been developed for MSI detection in colorectal and gastric cancers. These models used six and seven microsatellites (mono- and di-nucleotide repeat), respectively, and were based on a two-population (MSI vs. MSS) model in which the binomial distributions mixture was estimated by maximum likelihood using an expectation-maximization algorithm (55, 89).

**Figure 3:**
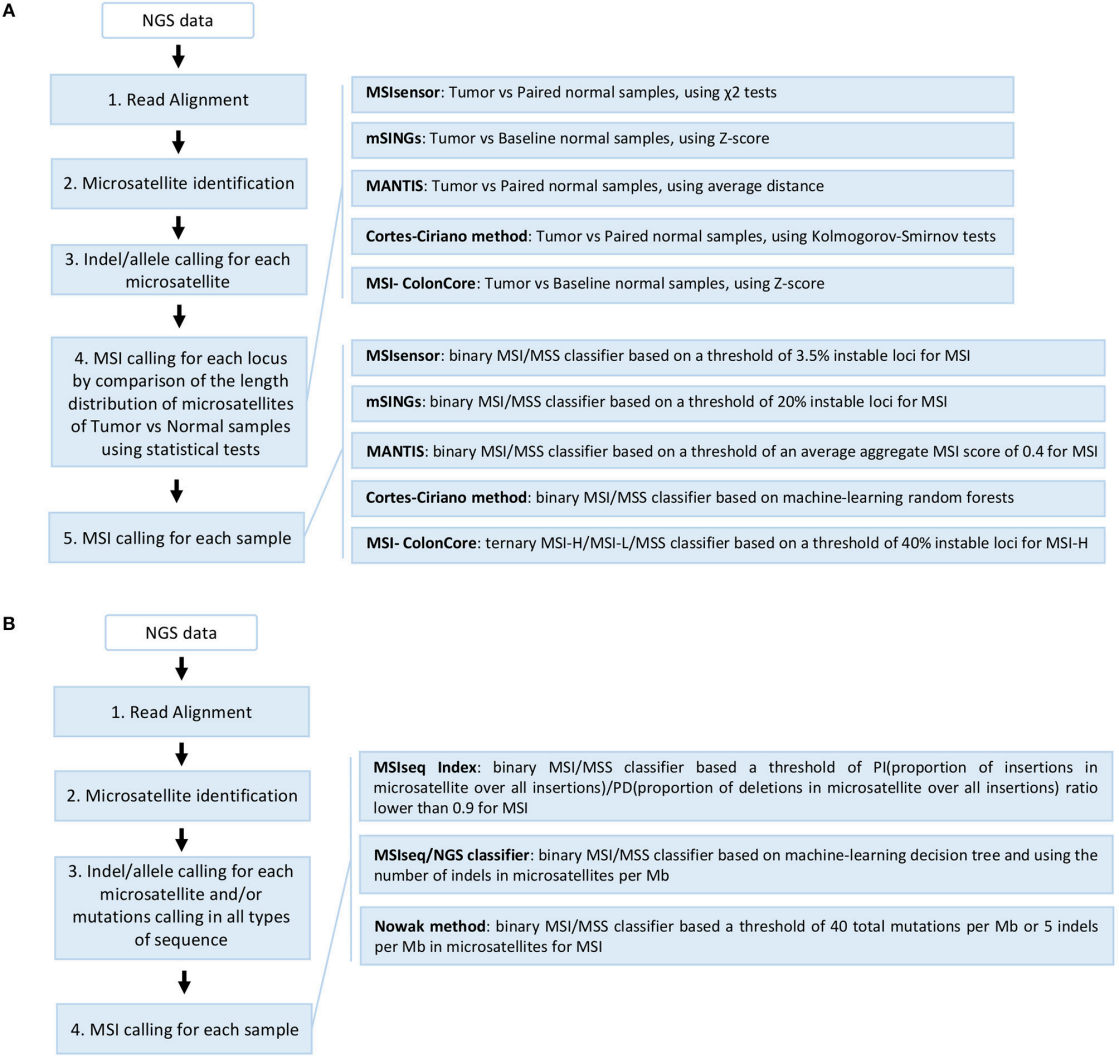
Overview of the different NGS-based computational methods developed for MSI detection in cancer. **(A)** Methods based on comparison of repeat length distribution of microsatellites including MSIsensor, mSINGs, MANTIS, Cortes-Ciriano method, and MSI-ColonCore. **(B)** Methods based on the total mutation burden in all sequences and/or the indel burden in microsatellites including MSIseq Index, MSIseq/NGS classifier, and Nowak methods. The steps 1–3 can be performed in different orders or in parallel depending on the method. The MSIseq/NGS classifier directly processes a list of somatic mutations and not raw NGS data.

Several computational approaches based on WGS, WES, and TGS data have been used to detect MSI, taking into account the difficulties of microsatellite sequences including management of stutter peak formation induced by PCR amplification during NGS library preparation, sequencing errors induced by homopolymers, and the shortcomings of sequencing read length which limits the length of the microsatellites analyzed (Figure 3).

The first study on colorectal and endometrial cancer described an MSI detection approach using WES and WGS data from The Cancer Genome Atlas (TCGA) (90). This study used mono- to tetra-nucleotide repeat microsatellites identified by Sputnik (91) to detect unstable microsatellites by comparing their length distribution between tumor and matched normal samples using the Kolmogorov-Smirnov statistic but without proposing a MSI/MSS tumor classifier (56).

Subsequently, different methods were developed for MSI detection in cancer, based on the comparison of the length distribution of a selection of microsatellites obtained by read count of all alleles (Figure 3A). For example, MSIsensor, a method which uses paired tumor and normal WES data to compare the allele length distribution of mono- to penta-nucleotide repeat microsatellites, and applies the chi square statistic for each locus analyzed, giving an MSI score that corresponds to the percentage of unstable microsatellite loci with a threshold of 3.5% for the MSI tumor phenotype (29). The second method developed was mSINGs, which uses WES and TGS data to investigate for MSI in 15–2957 mononucleotide repeat microsatellites, and compares the allele length distribution of each microsatellite in the MSI-negative control samples (baseline) with that of the tumor samples to detect unstable microsatellites; it uses a Z-score approach and a threshold of 20% of unstable markers to define MSI in tumors (27). This method has notably allowed the detection of MSI in 14 out of 18 types of cancer using TCGA data (17). Another method, MANTIS, uses a set of mono- to penta-nucleotide repeat microsatellites from WES data to detect MSI, by individually computing and aggregating the differences between the allele length distribution of each locus of matched tumor and normal samples to achieve an average distance score (0: fully stable and 2: fully unstable); a score threshold of 0.4 is recommended to diagnose MSI in tumors (28). MANTIS presents a higher overall sensitivity and specificity than MSIsensor and mSINGs, and has been used for MSI detection in 39 cancer types from TCGA (18). In a recent study based on WES and WGS data from TCGA, 23 cancer types were analyzed and unstable mono- to tetra-nucleotide repeat microsatellite loci were identified from tumor and normal samples by Kolmogorov-Smirnov statistics; the results were used to build a binary classifier based on the random forest model to predict MSI status (4). Finally, the MSI-ColonCore method was developed based on TGS data. In this method, the allele length distribution of mononucleotide repeat microsatellites from tumor samples are compared to a baseline formed by normal MSS reference samples using a Z-score approach to predict the MSI status of tumors, which are then classified into three possible groups: MSI/MSI-H, MSI-L, and MSS (48).

Other methods developed for MSI assessment in cancer use a different approach based on the mutation burden in all sequences and/or the burden of indels in microsatellites (Figure 3B). To date, MSI-seq Index is the only MSI detection method that is based on RNA sequencing data and the ratio of two measures, called PI and PD, which correspond to the proportion of insertions and deletions in mono- to hexa-nucleotide repeat microsatellites among all insertions and deletions found in RNA transcripts, respectively (92). When a threshold of 0.9 is applied, the PI/PD ratio is able to distinguish between MSS and MSI tumor samples without the use of matched normal samples (92). The MSI-seq/NGS classifier software offers a classifier for MSI assessment using WES somatic mutation data (small nucleotide substitutions and indels) and based on four machine-learning frameworks (logistic regression, decision tree, random forest, and naïve Bayes)(93). This method uses the rate and ratio of the small nucleotide substitutions in all sequences, and the indels of mono- to tetra-nucleotide repeat microsatellites, to classify the samples into “MSI” and “non-MSI”; it gives the best results with the decision tree classifier (93). Finally, the latest method to be developed uses targeted sequencing data from 275 genes implicated in cancer where the total mutation burden (>40 per Mb) and the indels in mononucleotide microsatellites (>5 per Mb) are used to define tumor samples such as MSI-H (94).

If the main advantage of these NGS based computational methods is that they allow a large number of microsatellite loci to be screened simultaneously, it should be remembered that NGS experiments are much more expensive to perform and require more time to generate results due to the more complex bioinformatics analysis required, and that they give similar or only slightly better sensitivity compared to standard capillary electrophoresis procedures (27, 46, 48).

Among the computational methods for MSI detection in cancer, MSIsensor (https://github.com/ding-lab/msisensor, v0.5, last updated on September 18, 2018) (29), mSINGs (https://bitbucket.org/uwlabmed/msings, v3.4, last updated on August 10, 2018) (27), MANTIS (https://github.com/OSU-SRLab/MANTIS, v1.0.4, last updated on June 19, 2018) (28), and MSIseq/NGS classifier (https://CRAN.R-project.org/package=MSIseq, v1.0.0, last updated on June 15, 2015) (93) are available online for download.

## Conclusion

Although the discovery of MSI in cancer is a quarter of a century old, it is relatively recent compared to the discovery of point mutations, epimutations, and copy number variations in cancer. The different methods developed so far for the detection and identification of MSI in cancer are mostly derived from methods created initially for the study of natural variations/polymorphisms present in the human genome or for point mutations present in cancer. These methods still appear to be in their infancy since to date only a small number have been developed for MSI detection, and of these very few are applicable to potential clinical applications of interest such as the detection of MSI in plasma, circulating tumor cells, blood, heterogeneous tumors, or tumors with a high level of normal cell contamination. In contrast, these methods have already been implemented for other types of alterations in cancer.

Numerous difficulties could explain these delays in technological development, notably the high polymorphism levels of microsatellite sequences in the different human populations and the errors induced by PCR slippage during amplification of microsatellite sequences, which increases the complexity not only of the analysis of microsatellite profiles but of the MSI detection and the identification of mutant alleles. More recently, several factors such as the errors induced during the sequencing-by-synthesis of homopolymers by next-generation sequencing, the difficulties of alignment of repetitive DNA sequences due to the short read length, and the low accuracy of indel calling, have also created a computational challenge for MSI detection by NGS-based computational methods, due to the high risk of interperation errors, a problem which requires a longer duration for the development of algorithms.

Given the recent discovery of MSI in dozens of types of cancer, which correlates in a positive dose-effect manner to survival outcome, and the demonstration that MSI is a major predictive biomarker for the efficacy of immune checkpoint blockade therapy in solid tumors, there is undoubtedly a need for the development of new sensitive tools for MSI detection, including clinical applications for MSI diagnosis. Future perspectives should include (i) the development of pan-cancer panels of highly sensitive and specific microsatellite markers for the detection of MSI in cancer, and (ii) the development of combined methods to improve the limit of detection of MSI, as required for certain types of clinical samples (i.e., tumors with high normal cell contamination, blood, and plasma); the latter could include the enrichment of mutant alleles prior to massively parallel NGS. These methods could then be rapidly implemented in routine clinical applications for the diagnosis of MSI in cancer.

## Author Contributions

LB, J-FD, and AH-K conceived, wrote and approved the final version of the submitted manuscript. AH-K supervised the whole writing of the manuscript.

### Conflict of Interest Statement

The authors declare that the research was conducted in the absence of any commercial or financial relationships that could be construed as a potential conflict of interest.
